# Librarian involvement on knowledge synthesis articles and its relationship to article citation count and Journal Impact Factor

**DOI:** 10.29173/jchla29798

**Published:** 2024-12-01

**Authors:** Krista Louise Alexander, Katharine Hall, Yuling Max Chen

**Affiliations:** 1 Reference & Subject Librarian, Concordia University Library, Montreal, QC; 2 Reference & Subject Librarian, Concordia University Library, Montreal, QC; 3 Department of Statistics & Actuarial Science, University of Waterloo, Waterloo, ON

## Abstract

**Introduction:**

Our aim was to determine if there is a relationship between librarian involvement on a knowledge synthesis project and the synthesis’s citation count or the Journal Impact Factor (JIF) of its publication venue.

**Methods:**

A total of 551 knowledge syntheses published during a one-year period (2020) from a single category, “Psychology, Clinical”, in Clarivate’s Journal Citation Reports were exported from Web of Science along with the citation counts for each synthesis and the JIF of its publication venue. The full-text of each article was examined in order to code each as either co-author, acknowledged, or unknown to reflect the level of librarian involvement in the synthesis. The Wilcoxon Rank Sum test on bootstrapped samples was used to determine the significance of the results.

**Results:**

Librarians were co-authors or acknowledged in 80 (15%) of the syntheses examined. Analyzing two levels of librarian involvement (involved, unknown) indicated no relationship between the level of librarian involvement and the JIF of the journal nor the citation count the synthesis received since publication.

**Discussion:**

There is no evidence of a relationship between librarian involvement in knowledge syntheses and the JIF of the publication or citation count of documents published in journals falling in the JCR category of “Psychology, Clinical” in the year 2020. Repeating this methodology in a different JCR category could help determine whether this lack of a relationship extends beyond the “Psychology, Clinical” category.

## Introduction

For more than a decade, guidance documents for carrying out systematic reviews and other knowledge syntheses have encouraged librarian involvement in the process of search strategy creation [1,2]. Gore and Jones provide an overview of methodology and guidance documents that reference the need for a librarian or expert searcher in their recommendations [3]. The benefits of librarian inclusion on systematic review teams have also been documented and include a lower risk of bias in systematic reviews that have librarian co-authors [4], improved search strategy reporting, search methods and search strategy quality [5-8]. Results of more recent research, including an investigation into systematic reviews associated with Vancouver hospitals and research centers, and an investigation into protocols and systematic reviews in education, have confirmed benefits of librarian involvement on search method quality, and alignment with search method and reporting standards [9,10].

Despite the clear advantages to including librarians on knowledge synthesis projects, research shows that involvement of librarians on systematic reviews can range from 0.66% to 63% [4-15]. Logan’s investigation into researcher motivations for including librarians as co-authors on evidence syntheses found that researchers that didn’t co-author or weren’t interested in coauthoring with librarians felt that “they already had the necessary expertise” [16]. This finding was echoed by Pawliuk et al.’s survey that found 75% of respondents that hadn’t involved a librarian in their systematic review “did not believe it was necessary to involve a librarian” [9]. It is important to keep in mind that librarian participation on knowledge synthesis projects are decisions that do not lie solely with the project’s principal investigator. In some contexts, librarians have the choice as to whether they accept this work. And indeed, when considering the time involved [17] and challenges associated with assisting on systematic reviews [18], arguments can certainly be made as to why librarians should be cautious about their level of involvement. It should be noted however, that only a single respondent to Pawliuk et al.’s survey indicated that the reason they did not involve a librarian on their systematic review was due to “being told the librarian was unable to help them” [9].

Given that past research [4-15] indicates that a large proportion of knowledge synthesis teams do not include or acknowledge a librarian, we were curious if there were other outcomes impacted by librarian presence that could be of interest to the faculty members and researchers that put these teams together. One outcome of potential interest is that of research impact metrics. There are many metrics that attempt to quantify research impact, but frequently employed metrics include article citation counts which are specific to the individual published article and the Journal Impact Factor (JIF) which focuses on the publication venue. Does librarian involvement on a knowledge synthesis project have an impact on where it is published or how frequently it is cited?

JIF is a proprietary metric calculated and published annually by Clarivate in their Journal Citation Reports (JCR) product. It is a ratio of the number of citations that items (published in the last two years) in a journal have received in a specific year, compared to the number of articles and reviews published in that journal for the last two years [19]. It is problematic to conflate JIF and citation counts with research quality [20], and it is important to keep in mind that these metrics are not without flaws. One example is the potential for JIFs to be manipulated, either by editors asking researchers to cite the journal in their submissions, or by citing it themselves in their own editorial pieces [21]. Even though such flaws have led to arguments being made against the use of JIFs in research assessment [22], it is still a factor of concern and consideration by researchers and academia. McKiernan et al. found that 40% of 57 research-intensive universities from across North America, “mentioned the JIF explicitly or used one of the JIF-related terms” [23] in their documentation related to promotion, tenure, and review. Accordingly, faculty members concerned with publication profile, research impact, and evaluation, may be influenced by the prospect of publishing in a higher impact factor journal. In Niles et al.’s online survey, JIF was identified as one of the top criteria that researcher respondents thought their peers valued when it came to selection of publication venue [24]. Even if researchers do not value JIFs themselves, their perception of what their peers value can still be an influential factor, especially in institutions that have a peer-based evaluation system. If librarian involvement on a knowledge synthesis project led to a published synthesis that received a higher number of citations or was published in a journal with a higher JIF, it could be an additional incentive for faculty members to include librarians on their synthesis teams.

Librarian involvement and its relationship to citation count and JIF is an idea that has been briefly posited as an area for future research by Ross-White in her 2016 investigation into librarian involvement on systematic reviews at Queen’s University [11]. Three groups have since investigated the relationship between librarian involvement on systematic reviews and JIF or citation count. In 2022, Wang and Lin found a statistically significant higher mean citation count for systematic review articles without librarian involvement [13]. It should be noted however, that no indication of disciplinary spread was provided for their sample in the English language summary of this Chinese language article. A poster from Craven, Palmer, and Piper focused on systematic reviews published by researchers at the University of Colorado Anschutz Medical Campus and found that comparison of reviews with and without librarian co-authors showed “no statistical difference between groups regarding the JIF of journals” [25]. It is important to note that their analysis of librarian co-authorship appears to have been based on their recognition of author names provided in citations, so would not have accounted for librarian coauthors beyond their institution – a fact they point to on their poster as being one limitation of their research. While a more detailed explanation may have been given when they presented this research, their poster does not provide the disciplinary spread of the articles in their sample, making it hard to determine if their comparative analysis based on JIF is appropriate. They did account for disciplinary differences in citation rates by performing an analysis of the percentile rank of the JIF of journals in their study, however they still found no statistically significant difference in mean percentile rank between those articles with and without librarian co-authors [25]. In 2023, another group identified the level of librarian involvement on 280 systematic reviews published in dentistry journals. They too found no statistically significant difference in mean JIFs for reviews that involved a librarian (either as a co-author or consultant) and those that did not [14]. However, this study used a one-year publication period that straddled two calendar years (July 1, 2018 – July 1, 2019) and it is unclear how or whether differences in 2018 and 2019 JIFs for each journal were accounted for in their analysis.

In contrast to these three studies, we chose to focus our research on knowledge synthesis articles published within a single calendar year, 2020, as it was the most recent edition of JCR available when we began this research. Different disciplines have different citation habits, making comparison of JIFs between journals from different disciplines problematic [21]. For this reason, our knowledge synthesis sample was restricted to documents published in journals from a single subject category in JCR [26]. The category of “Psychology, Clinical” was selected. Our institution, Concordia University, does not have a medicine, dentistry, or nursing program. Focusing on a discipline (psychology) known for producing a large portion of the systematic reviews at our institution [27] made more sense than focusing on an area of medicine (perhaps traditionally more commonly associated with systematic reviews).

## Methods

### Knowledge synthesis sample

Our sample set of knowledge synthesis articles was obtained by constructing a search for Clarivate’s Web of Science Core Collection [28]. We took inspiration from the systematic reviews search filter used in PubMed [29] to generate keywords. The final search strategy contained three components:

1. Keywords to identify knowledge syntheses, including systematic reviews, scoping reviews, rapid reviews, umbrella reviews, realist reviews, mapping reviews or meta-analyses.
2. ISSN and/or eISSN of publications from JCR’s “Psychology, Clinical” category.
3. Publication year of 2020

The complete search strategy for Web of Science can be found in the Online Supplement, Appendix – Search strategy.

**Fig. 1:**
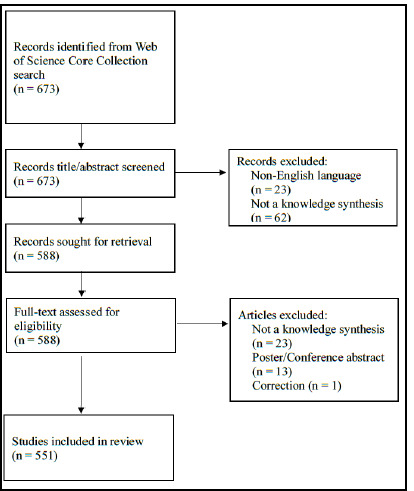
PRISMA flow chart for identification of knowledge syntheses

The search retrieved 673 results, which were imported into the systematic review software Rayyan [30]. As the records came from a single database, duplicates were not expected, but Rayyan was used to confirm that this was indeed the case. All title and abstracts were screened independently by both authors to ensure that they were knowledge syntheses, with all conflicts resolved through discussion. We took guidance from the definition put forth by Meert, Torabi and Costella and considered any review identified by its authors as a systematic review, scoping review, rapid review, umbrella review, realist review, mapping review, meta-analysis (with data derived from a literature search) or those studies containing a systematic-style literature search as part of the methodology [7]. Removed from the sample were protocols, meeting or conference abstracts, poster abstracts, narrative reviews, meta-analyses where the data was not derived from a literature search, and non-English language articles. Full-text retrieval was completed for 588 syntheses. Additional exclusions were made during the full-text review leaving a total of 551 syntheses in our dataset. The flow of records through these stages is illustrated in Figure 1.

Supplement, Appendix 1

### Level of librarian involvement

These 551 documents were analyzed and categorized independently by each author to determine if there was participation of a librarian on the synthesis, with all conflicts being resolved through discussion. Documents were placed into one of three categories, based on the level of librarian involvement:

- Co-author: librarian listed as co-author on the review
- Acknowledgement: librarian(s) mentioned (by name or profession) in either the body of the text or the acknowledgements section of the review
- Unknown: no authors or acknowledged individuals are librarians, or unable to confirm if authors or acknowledged individuals are librarians

To determine the Library and Information Science (LIS) credentials of the individuals associated with the review, we followed similar methods to those used by Rethlefsen et al. [6] as they allowed us to create a complete-as-possible dataset without individually surveying or contacting the authors. The author list of each article in our sample was assessed for the presence of LIS degrees and/or an affiliation with a library or information centre. When neither degrees nor departmental affiliations were listed in the author list, we searched institutional directories and Google to determine the potential LIS status of the individuals associated with the review. If there were no librarians listed as authors, the body of the article and the acknowledgement section were scrutinized for the mention of “librarian” or “information scientist” or “information specialist” or “informationist.” If individuals were named in the acknowledgements section, but with no indication of their profession or role, their names were searched to determine their LIS status. Conflicts arose in instances where evidence of LIS status was found by one author but not the other in their searches. These conflicts were resolved by discussion of that evidence with the second author.

### Statistical methods

Both the JIFs and citation counts are numerical values that do not quite meet the definition of continuous data. The distribution of both JIFs and citation counts are also not normal. Instead of the well-known t-test, we chose to use the non-parametric alternative known as the Wilcoxon Rank Sum test [31], with significance level (alpha) threshold of 0.05. A p-value larger than 0.05 would indicate no statistical evidence in the data to support the claim.

Upon initial analysis of the results, we discovered that there was a relatively small number of knowledge syntheses co-authored by librarians.

We combined the groups of Co-author and Acknowledged into one group, and renamed it Librarian Involved, in hope of obtaining two moderately balanced groups. However, even with the combined group of Librarian Involved, the dataset groups remained unbalanced. Further analyses using the imbalanced data may lead to biased models and inaccurate predictions. To account for this, we employed the bootstrap method [32]. This approach involves iteratively and randomly sampling both groups (Librarian Involved and Unknown Involvement) to create smaller, balanced samples in order to compare their corresponding JIFs and citation counts. This random sampling process is repeated 500 times with 50 random samples from each group.

## Results

### Level of librarian involvement

**Fig. 2:**
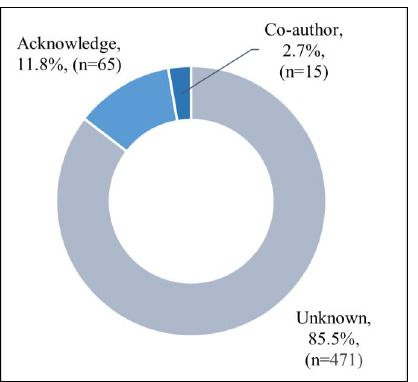
Level of librarian involvement in knowledge synthesis sample (n=551)

Of the 551 knowledge syntheses included in this study, 15% (n=80) had a credited level of librarian involvement, with 3% (n=15) having a librarian coauthor and 12% (n=65) acknowledging a librarian (see Figure 2).

Tables 1 and 2 contain the mean, median, minimum and maximum JIF and article citation count for each level of librarian involvement.

**Table 1:** Descriptive statistics of Journal Impact Factor based on level of librarian involvement

	Co-Author (n=15)	Acknowledged (n=65)	Unknown (n=471)	All (n=551)
Median JIF	4.38	4.33	4.52	4.51
Mean JIF	5.51	4.97	5.48	5.42
Lowest JIF	3.23	1.61	0.34	0.34
Highest JIF	12.79	12.79	12.79	12.79

**Table 2:** Descriptive statistics of knowledge synthesis citation counts (as of Sept. 6, 2024) based on level of librarian involvement

	Co-Author (n=15)	Acknowledged (n=65)	Unknown (n=471)	All(n=551)
Median citation count	15	21	26	26
Mean citation count	38.9	30.6	40	38.6
Lowest citation count	0	2	0	0
Highest citation count	233	311	253	311

**Fig. 3:**
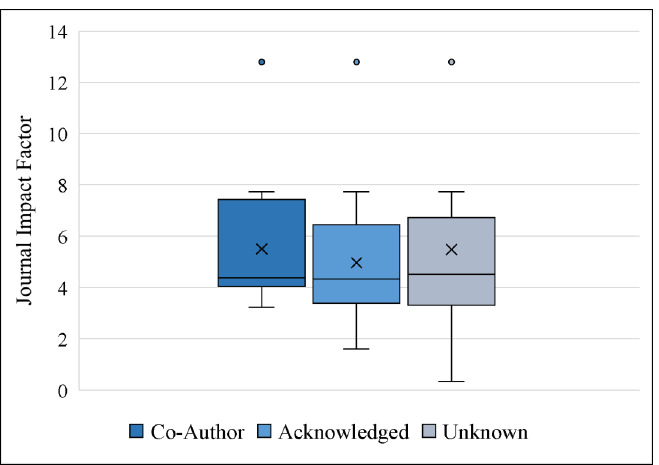
Box and whisker plot of JIFs by level of librarian involvement. The X shows the group mean, the bar shows the median.

**Fig. 4:**
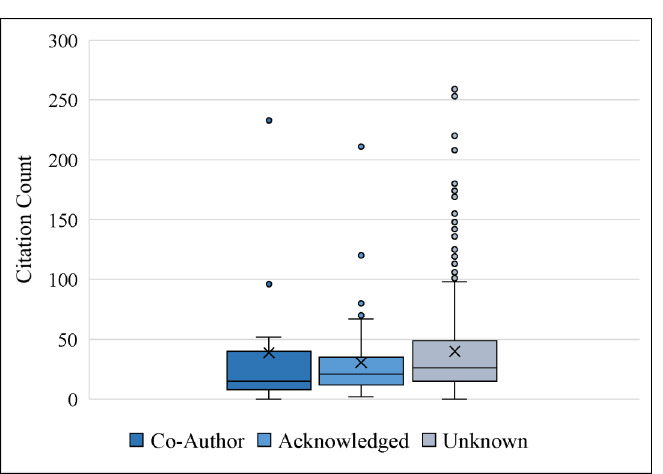
Box and whisker plot of citation counts (as of Sept. 6th 2024) by level of librarian involvement. The X shows the group mean, the bar shows the median.

Figures 3 and 4 display the distribution of JIFs and article citation counts by librarian involvement. Both figures show that the means (marked with an X) are greater than the medians (marked with a bar). This is an indication that the data are not normally distributed. In fact, they are positively skewed, i.e. most articles have low JIFs or citation counts, with a few exceptions.

### Librarian impact on Journal Impact Factor and citation count

To understand the relationship between librarian involvement, JIFs, and citation count, we used the Wilcoxon Rank Sum two-sided test with bootstrapping as mentioned in the Methods section. The bootstrapped samples, on average, suggest no evidence that librarian involvement has an impact on the JIF of the publication venue (W = 1196.16, p = .61). This is not surprising because the medians (see Figure 3) are similar, and the standard deviations are large. Similarly, the bootstrapped samples also suggest no evidence that librarian involvement has any impact on the number of citations received by an article (W = 1045.54, p = .16). Figure 4 shows that the medians are quite close to each other, whereas the spread of the data (standard deviation) is large.

## Discussion

### Level of librarian involvement

Previous studies that have reported on librarian involvement in systematic reviews found a wide range that spans 0.66% to 63% [4-15]. While we found levels of co-authorship (3%) and librarian acknowledgement (12%) that were low compared to the syntheses with unknown librarian involvement (85%), it fell within the range found in other studies.

It is important to note however, that there were distinct differences between studies with respect to discipline, year range, and synthesis type (i.e., systematic review versus other forms of knowledge syntheses), making it hard to do direct comparisons of results. Several studies were exclusively considering reviews that were authored or co-authored by researchers at specific institutions [4,11,15], while others drew a sample set from a single JCR subject category [6,7]. For example, Rethlefsen et al. reported a librarian involvement rate of 23.3% acknowledgement and 7.1% co-authorship, but their research was limited to systematic reviews published in top JIF journals from the General and Internal Medicine subject category [6]. Similarly, Meert, Torabi, and Costella reported 11% librarian involvement but were examining systematic reviews published in top JIF paediatrics journals [7]. Beyond obvious differences in discipline, if only top JIF journals were examined by these other research teams, it is unknown what the rate of librarian involvement was across the entire JIF range of the journals in the JCR categories examined in their studies.

Although the levels of librarian involvement quoted here make it appear that different disciplines involve librarians in their knowledge synthesis projects more frequently than others, differences in year range examined and synthesis type, amongst other factors, could also have played a role. As such, this could serve as an interesting area for further study.

### Librarian impact on Journal Impact Factor

Statistical analysis of our data showed no evidence of a relationship between librarian involvement on a knowledge synthesis project and the JIF of the journal in which that synthesis is published for the JCR category of “Psychology, Clinical” in 2020. Although statistical methods were used to compensate for the relatively low proportion of syntheses that involved a librarian, an interesting avenue for future research would be to examine a JCR category with a higher proportion of librarian involvement. The proportion of articles involving a librarian would be unknown until an analysis was complete, however, previous research could potentially be used to inform the initial choice of JCR category. Another option would be to examine knowledge syntheses from journals that fall into different JCR categories, but use the rank of each journal (based on JIF) in their respective JCR category, for analysis, as was done by Craven, Palmer, and Piper in their poster [25].

### Librarian impact on citation count

An analysis of each individual document’s citation performance was carried out to determine if there was a relationship between citation count and librarian involvement on knowledge syntheses published in “Psychology, Clinical” journals in 2020. Statistical analysis showed no evidence of a relationship; librarian involvement on a knowledge synthesis project does not appear to lead to a higher or lower number of citations to that synthesis. Wang and Lin had previously reported that systematic reviews without librarian involvement received, on average, a higher number of citations than systematic reviews with librarian involvement [13]. With our only access to their research being an abbreviated English language summary of their article, we are unable to assess the specific parameters of their methodology and findings, but these conflicting results suggest that perhaps it is an area that warrants further exploration.

### Limitations

In addition to the limitations mentioned above, we acknowledge that other limitations were present in the methodology. Non-English language articles were excluded from our sample, as analysis of certain portions of the article were necessary to determine the level of librarian involvement and both authors only speak English. We also made the decision to exclude conference proceedings, meeting abstracts, and posters, even though they may describe synthesis research that includes librarians. Our decision to exclude these research outputs was because they are not subjected to the same editorial and peer-review processes as knowledge synthesis manuscripts. And indeed, only articles and reviews are counted as part of a journal’s document count when it comes to calculating its JIF [19]. Protocols were excluded from our analysis. Research has shown that even if a protocol declares an intention to consult a librarian, it does not always lead to a librarian acknowledgement in the final synthesis, making it unclear whether that consultation ever took place [10]. Not all journals with JIFs in the JCR category “Psychology, Clinical” published systematic reviews in the time period under consideration, which meant that only a subset of the category’s journals were actually analyzed. The presence of international authors on the documents made identification of librarians challenging. Although the term librarian is used to signal this profession in Canada and the United States, the terms librarian, information specialist, documentation technician etc. could potentially indicate different roles in different countries. Different degrees beyond library or information science may be sufficient for work as a librarian in other countries as well. Where there were conflicts as to the level of librarian involvement or the LIS status of an individual, we came to a consensus through discussion, but we recognize that this method is not without its limitations. It is important to also keep in mind that the knowledge syntheses categorized as having an unknown level of librarian involvement, may have consulted with a librarian without acknowledging them in the published article. This was the case in Meert, Torabi, and Costella’s research where they found 11% librarian involvement based off of examination of articles, but found 22% librarian involvement when article examination was combined with survey responses from authors about whether a librarian had been involved in the project [7].

Using a synthesis’s citation count for this analysis was also not without limitations. Given that our analysis was based on a full year’s worth of publications and the citation information was extracted for all the articles once (September 6, 2024), those syntheses published in January 2020 would have had nearly an additional year to accumulate citations, compared to those published in December 2020.

## Conclusion

An analysis of all knowledge syntheses published in 2020 in titles from the JCR category “Psychology, Clinical” found that 3% were co-authored by librarians and 12% acknowledged a librarian in the text of the syntheses. There was no evidence of a relationship between librarian involvement in those knowledge syntheses and the Journal Impact Factor of the final publication venue or in the number of citations those syntheses received.

## Data Availability

Due to its proprietary nature, the data collected and analyzed in this study is available upon request only.
