# Supplementary material for: Librarian involvement on knowledge synthesis articles and its relationship to article citation count and Journal Impact Factor
Source: J Can Health Libr Assoc. 2024 Dec 1;45(3):137–46. doi: 10.29173/jchla29798 (PMC11881647; doi:10.29173/jchla29798)
Supplement: Supplementary file 1 [file JCHLA-45-137-s001.pdf]

Appendix – Search strategy

The following search was developed to identify systematic reviews, scoping reviews, rapid reviews, umbrella reviews, realist reviews, mapping reviews or meta-analyses published in 2020 in the Social Sciences Citation Index Category of "Psychology, Clinical". Search performed on Clarivate's Web of Science interface.

((TI=(((systematic OR scoping OR rapid OR umbrella OR realist OR mapping) NEAR/2 review) OR ((Knowledge OR evidence) NEAR/2 (synthesis OR syntheses)) OR (meta-analysis OR meta-analyses) OR "overview of reviews" OR ("review" NEAR/2 "reviews") OR (review NEAR/2 meta*))) OR (AB=(((systematic OR scoping OR rapid OR umbrella OR realist OR mapping) NEAR/2 review) OR ((Knowledge OR evidence) NEAR/2 (synthesis OR syntheses)) OR (meta-analysis OR meta-analyses) OR "overview of reviews" OR ("review" NEAR/2 "reviews") OR (review NEAR/2 meta*)))) NOT (TI=(protocol OR protocols))

AND FPY=(2020)

AND (IS=(1548-5943 OR 0272-7358 OR 1873-7811 OR 0033-2917 OR 1469-8978 OR 1040-7308 OR 1573-6660 OR 1743-7199 OR 1743-7202 OR 2167-7026 OR 2167-7034 OR 0969-5893 OR 1468-2850 OR 0021-843X OR 1939-1846 OR 0033-3204 OR 1939-1536 OR 1091-4269 OR 1520-6394 OR 1740-1445 OR 1873-6807 OR 1650-6073 OR 1651-2316 OR 1096-4037 OR 1573-2827 OR 1697-2600 OR 1576-7329 OR 0022-006X OR 1939-2117 OR 1553-2739 OR 1434-4599 OR 0887-6185 OR 1873-7897 OR 0022-4499 OR 1559-8519 OR 1040-3590 OR 1939-134X OR 1537-4416 OR 1537-4424 OR 0276-3478 OR 1098-108X OR 1868-8527 OR 1868-8535 OR 1073-1911 OR 1552-3489 OR 1072-4133 OR 1099-0968 OR 0004-

0002 OR 1573-2800 OR 0005-7967 OR 1873-622X OR 0160-6689 OR 1555-2101 OR 2214-7829 OR 0963-8237 OR 1360-0567 OR 0278-6133 OR 1930-7810 OR 0005-7894 OR 1878-1888 OR 2152-0828 OR 2152-081X OR 0144-6657 OR 2044-8260 OR 2000-8198 OR 2000-8066 OR 2050-2974 OR 2050-2974 OR 1476-0835 OR 2044-8341 OR 0306-4603 OR 1873-6327 OR 1041-6102 OR 1741-203X OR 1098-3007 OR 1538-4772 OR 0091-0627 OR 1573-2835 OR 1557-1874 OR 1557-1882 OR 0095-2990 OR 1097-9891 OR 0022-3891 OR 1532-7752 OR 0740-5472 OR 1873-6483 OR 1050-3307 OR 1468-4381 OR 0021-8855 OR 1938-3703 OR 1949-2715 OR 1949-2723 OR 1385-4046 OR 1744-4144 OR 0014-7370 OR 1545-5300 OR 1079-0632 OR 1573-286X OR 0894-9867 OR 1573-6598 OR 0145-4455 OR 1552-4167 OR 1359-107X OR 2044-8287 OR 0894-4105 OR 1931-1559 OR 1359-1053 OR 1461-7277 OR 1942-9681 OR 1942-969X OR 1064-0266 OR 1532-530X OR 1064-1297 OR 1936-2293 OR 2212-1447 OR 2212-1455 OR 0160-7715 OR 1573-3521 OR 0167-482X OR 1743-8942 OR 1077-7229 OR 1878-187X OR 1179-1578 OR 1179-1578 OR 1063-3995 OR 1099-0879 OR 0021-9762 OR 1097-4679 OR 2520-8969 OR 2520-8977 OR 0198-7429 OR 2163-5307 OR 0887-6177 OR 1873-5843 OR 0093-8548 OR 1552-3594 OR 0092-623X OR 1521-0715 OR 1529-9732 OR 1529-9740 OR 0279-1072 OR 2159-9777 OR 1471-0153 OR 1873-7358 OR 0893-3200 OR 1939-1293 OR 1541-1559 OR 1939-148X OR 0882-2689 OR 1573-3505 OR 1068-9583 OR 1573-3572 OR 0090-5550 OR 1939-1544 OR 0002-7642 OR 1552-3381 OR 1359-1045 OR 1461-7021 OR 0147-5916 OR 1573-2819 OR 1380-3395 OR 1744-411X OR 0194-472X OR 1752-0606 OR 1352-4658 OR 1469-1833 OR 1070-5503 OR 1532-7558 OR 1053-8712 OR 1547-0679 OR 0005-7916 OR 1873-7943 OR 0885-7482 OR 1573-2851 OR 1475-357X OR 1475-3588 OR 1092-6771 OR 1545-083X OR 1090-0586 OR 1573-3270 OR 2043-8087 OR 0736-7236 OR 1931-7611 OR 1931-762X OR 1072-0847 OR 1099-

078X OR 0932-8114 OR 1438-9460 OR 0894-9085 OR 1573-6563 OR 1550-4263 OR 1550-4271 OR 2512-8442 OR 2512-8450 OR 1328-4207 OR 1742-9552 OR 0736-9735 OR 1939-1331 OR 0813-4839 OR 2049-7768 OR 0937-2032 OR 1439-1058 OR 0163-4445 OR 1467-6427 OR 0197-4556 OR 1873-5878 OR 1533-7731 OR 1533-7731 OR 1468-1994 OR 1468-1749 OR 1752-2439 OR 1752-2447 OR 1937-1209 OR 1937-1217 OR 1072-0537 OR 1521-0650 OR 1132-9483 OR 1132-9483 OR 0002-9157 OR 2160-0562 OR 0020-7144 OR 1744-5183 OR 2578-8086 OR 2578-8094 OR 2183-6051 OR 2183-6051 OR 0012-1924 OR 2190-622X OR 0731-7107 OR 1545-228X OR 0192-6187 OR 1521-0383 OR 1016-6262 OR 1423-0402 OR 1534-6501 OR 1552-3802 OR 1438-3608 OR 2196-8349 OR 0935-6185 OR 1432-2080 OR 1130-5274 OR 2174-0550 OR 0718-4808 OR 0718-4808 OR 0020-7284 OR 1943-2836 OR 0889-8391 OR 1938-887X OR 1616-3443 OR 2190-6297 OR 2360-0853 OR 1661-4747 OR 1664-2929 OR 0017-4947 OR 2196-7989 OR 2689-5269 OR 2689-5277))

Note about Journal ISSNs:

The subject categories in JCR contain journals indexed in Clarivate's citation indexes (the Science Citation Index Expanded (SCIE), Social Science Citation Index (SSCI), Arts and Humanities Citation Index (AHCI) and the Emerging Sources Citation Index (ESCI)). For example, the JCR category of "Psychology, Clinical" (the category selected for this research) corresponds to a set of journals indexed in Clarivate's SSCI and ESCI.

Although the "Psychology, Clinical" category in the 2020 edition of JCR has 174 journal titles total, only 130 of those have been assigned Journal Impact Factors for 2020. It is these 130 journal ISSNs and/or eISSNs that were used for the Web of Science search. The remaining 44 titles consisted of 43 titles classified as part of the Emerging Sources Citation Index (ESCI) that

did not have 2020 Journal Impact Factors. One other title from the Social Science Citation Index (SSCI) also did not have a 2020 Journal Impact Factor (International Journal of Transgender Health). This appears to be a result of a title change that occurred in 2020. To account for this, this journal's ISSN and eISSN were added to the search, and any articles published in 2020 under the new journal title retrieved during the search will be assigned the 2020 Journal Impact Factor of the legacy title (International Journal of Transgenderism).
